# Monocytes conditioned media stimulate fibronectin expression and spreading of inflammatory breast cancer cells in three-dimensional culture: A mechanism mediated by IL-8 signaling pathway

**DOI:** 10.1186/1478-811X-10-3

**Published:** 2012-02-10

**Authors:** Mona M Mohamed

**Affiliations:** 1 Department of Zoology, Faculty of Science, Cairo University, Giza, 12613, Egypt

**Keywords:** Fibronectin, E-cadherin, IL-8, inflammatory breast cancer, monocytes

## Abstract

**Background:**

Inflammatory breast cancer (IBC) is the most aggressive form of breast cancer characterized by invasion of carcinoma cells into dermal lymphatic vessels where they form tumor emboli over expressing adhesion molecule E-cadherin. Although invasion and metastasis are dynamic processes controlled by complex interaction between tumor cells and microenvironment the mechanisms by which soluble mediators may regulate motility and invasion of IBC cells are poorly understood. The present study investigated the effect of media conditioned by human monocytes U937 secreted cytokines, chemokines and growth factors on the expression of adhesion molecules E-cadherin and fibronectin of human IBC cell line SUM149. Furthermore, cytokines signaling pathway involved were also identified.

**Results:**

U937 secreted cytokines, chemokines and growth factors were characterized by cytokine antibody array. The major U937 secreted cytokines/chemokines were interleukin-8 (IL-8) and monocyte chemotactic protein-1 (MCP-1/CCL2). When SUM149 cells were seeded in three dimensional (3D) models with media conditioned by U937 secreted cytokines, chemokines and growth factors; results showed: 1) changes in the morphology of IBC cells from epithelial to migratory spindle shape branched like structures; 2) Over-expression of adhesion molecule fibronectin and not E-cadherin. Further analysis revealed that over-expression of fibronectin may be mediated by IL-8 via PI3K/Akt signaling pathway.

**Conclusion:**

The present results suggested that cytokines secreted by human monocytes may promote chemotactic migration and spreading of IBC cell lines. Results also indicated that IL-8 the major secreted cytokine by U937 cells may play essential role in fibronectin expression by SUM149 cells via interaction with IL-8 specific receptors and stimulation of PI3K/Akt signaling pathway.

## Background

Inflammatory breast cancer (IBC) is the most lethal form of breast cancer associated with particularly aggressive behavior and poor prognosis in young women [1]. IBC is pathologically defined as invasive adenocarcinoma, where carcinoma cells possess high metastatic properties and ability to invade lymphatic vessels of breast stroma and skin forming tumor emboli [2]. Spreading of tumor emboli within lymphatic and blood vessels leads to distant metastasis and multi-organ failure in IBC patients [3].

Alterations in the expression of adhesion molecules such as the epithelial marker E-cadherin and the mesenchymal marker fibronectin [4] were found to play a crucial role in the progression of breast cancer metastasis [5-7]. IBC is characterized by over-expression of E-cadherin, a cell surface adhesion protein which mediates cell-cell contact [8]. Loss of E-cadherin in primary breast carcinoma was associated with disease poor prognosis [9]. Paradoxically, E-cadherin over-expression in IBC contributes to disease aggressiveness and low survival rate [8] since, E-cadherin expression by IBC carcinoma cells allows cell to cell adhesion and the formation of tumor emboli within the lymphatic vessels [10,11]. Moreover, the process of invasion and dissemination of IBC tumor emboli is mediated by expression of E-cadherin and the activity of matrix metalloproteinases (MMP-1 and MMP-9) [12]. E-cadherin has also been reported to be involved in different cellular biological processes including cell growth [13] and differentiation [14]. Furthermore, IBC cell lines SUM149 were found to express mesenchymal extracellular matrix (ECM) glycoprotein fibronectin [15] an adhesion molecule involved in cell-cell and cell-matrix adhesion [16]. Fibronectin is also associated with cell differentiation, oncogenic transformation, motility and migration [16]. For example, studies demonstrated that fibronectin increases the secretion of matrix metalloproteinase-9 (MMP-9) in ovarian cancer and stimulate the growth of non-small cell lung carcinoma via PI3K/Akt signaling pathway [17,18]. Interestingly, PI3K/Akt pathways found to induce fibronectin expression assuming a reciprocal stimulation of fibronectin production via PI3K/Akt pathway [19,20]

One of the limitations in understanding IBC biology may be due to the lack of an *in-vitro *culture model that simulates *in-vivo *tumor microenvironment. Studies showed that mammary tumor cells grown in monolayer or 2 dimensional (2D) culture exhibited different physiological and molecular properties than those grown in 3 dimensional (3D) cultures [21]. The *in vitro *3D culture provides mammary epithelial cells with basement membrane-like matrices that mimic *in vivo *growth. Growing cells in 3D models allow cell-matrix interactions that reorganize and modulate cytoskeleton, chromatin structure and cell polarity [22-24]. Furthermore, 3D culture can preserve cell physiological functions which cannot be carried out in monolayer culture. For example, mammary epithelial cells grown on 3D model produce casein [25] and hepatocytes can synthesize cytochrome P450 [26]. Focusing on the application of 3D culture models on breast cancer research there are various studies by Bissell and her collaborators showing that 3D cell culture assays could be used to study mechanisms for morphogenesis, gene/protein expression profiles and neoplasia of human breast *in vitro *[27-29]. For instance, Bissel and her colleagues compared the morphological phenotype and gene expression profile of 25 breast cancer cell lines seeded in monolayer and in 3D culture. Their results showed that breast cancer cell lines cultured in 3D form "colonies" that could be morphologically divided into four groups: round, mass, grape like and stellate [29]. Moreover, gene expression profile and signal transduction pathways were different among cells when cultured in 3D versus monolayer culture [29]. Using *in vitro *3D models Hoffmeyer and colleagues compared the growth of IBC cell line SUM149 and non-IBC cell line SUM102 as control (since it shares SUM149 loss in inflammatory breast cancer gene) [30]. The SUM149 cells showed an increase in expression of Rho A and E-cadherin proteins and more adhesion to collagen I than SUM102. Over-expression of RhoA and E-cadherin may promote cell to cell adhesion which is essential for passive metastasis by IBC tumor emboli [30].

Dissemination of carcinoma cells is modulated by adhesion molecules, which may be affected by different factors including cues from inflammatory cells within the tumor microenvironment [31]. Monocytes/macrophages are the major inflammatory cells constituting breast tumor microenvironment [32,33] and contributing to high levels of growth factors, hormones, and cytokines [34,35]. Monocytes/macrophages secreted cytokines, chemokines and growth factors were found to induce the migration, invasion, and metastasis of carcinoma cells [36,37]. Furthermore, monocytes/macrophages secretions found to alter the expression of adhesion molecules fibronectin and E-cadherin. For instance, TNF-α, IL-1β, and IL-6, secreted by monocytes/macrophages were shown to modulate the expression of fibronectin by primary keratinocytes in 3D tissue culture models [38]. Similarly TNF-α was found to modulate expression of E-cadherin by bronchial epithelial cells [19]. On the other hand, oncostatin M released by activated mononuclear cells increased the expression of fibronectin and decreased the expression of E-cadherin by myofibroblasts [39]. A recent study demonstrated that interaction of IL-8 with its specific receptors CXCR1 and CXCR2 induce fibronectin expression essential for epithelial mesnchymal transition and motility of human breast carcinoma cells [40]. Thus, monocytes/macrophages secreted cytokines, chemokines and growth factors may modulate the expression of adhesion molecules E-cadherin and fibronectin by different cell lines.

In a previous study by Mohamed and colleagues, co-culture of SUM149 cells with U937 cells or in U937-conditioned media (U937-CM) in 3D tissue culture model increased invasiveness, motility and proteolytic activity of SUM149 cells [41]. Herein, the study aimed to identify secreted cytokines, chemokines and growth factors in U937-CM and to utilize 3D model to test whether invasion and motility of SUM149 in response to U937-CM may be also modulated by adhesion molecules E-cadherin and fibronection. Results indicated that U937-CM enhances the motility and spreading of SUM149 cells by increasing fibronectin expression. Moreover, IL-8 a major cytokine secreted by U937 cells found to be involved in fibronectin expression via stimulating PI3K/Akt signaling pathway.

## Results

### Formation of emboli like structure by SUM149 cells when grown on 3D culture model

The basic principle of 3D cell cultures is to provide mammary carcinoma cells with properties that recapitulate *in-vivo *tumor microenvironment. The 3D cellular structures were characterized by establishment of adhesion complexes, tissue polarity, cytoskeletal structures, and cell volume that is significantly different from those found in traditional monolayer culture [24,42].

**Figure 1:**
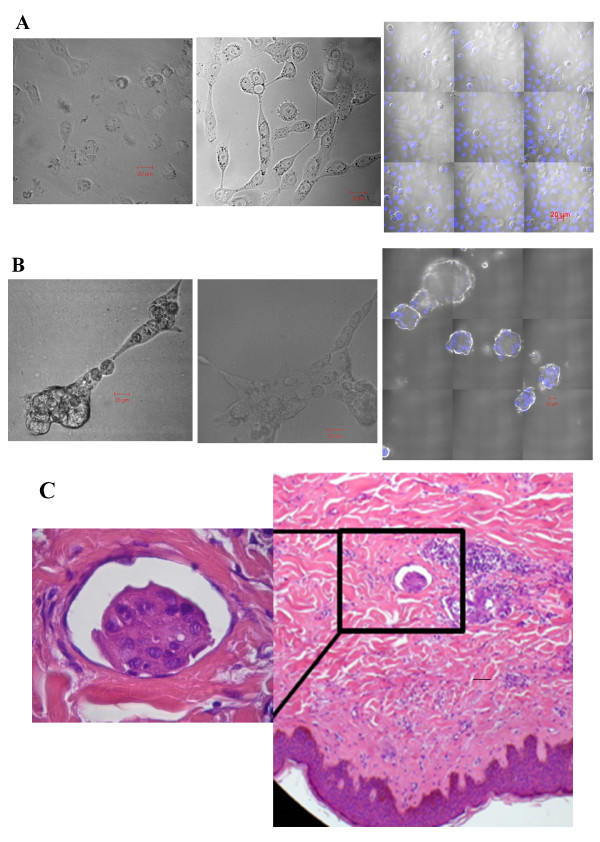
**Alteration in the morphology of SUM149 cells cultured in monolayer versus 3D culture models**. **A) **SUM149 cells grown on monolayer culture for 24 h showing cells adhering to monolayer (left panel). SUM149 cells divided and became attached to each other after 48 h (middle panel) reaching 80% confluent after 72 h as shown in 9 fields' image (right panel. Nuclei were stained with blue stain DAPI. **B) **SUM149 cells grown in 3D overlay culture cells form aggregates after 24 h (left panel), divide and form connected like structures after 48 h (middle panel). After 72 h cell division increased and SUM149 cells formed emboli like structures which were distributed all over 3D culture model shown in 9 fields image (right panel) nuclei stained with blue stain DAPI were distributed within the tumor emboli structures, (scale bar = 20 μm). **C) **Archival H&E stain of paraffin embedded tissue section of IBC patient sample showing IBC cells invasion into blood and lymphatic vessels forming tumor emboli while tumor cells strongly bound together and retracted away from surrounding endothelial lining. Results were representatives of 3 independent experiments.

Alteration in the morphology of SUM149 grown on monolayer versus those grown on 3D models were observed after 24 h, 48 h and 72 h. SUM149 cells grown on monolayer adhere to plastic surface after 24 h (Figure 1A, left panel). SUM149 cells form projections towards each other after 48 h (Figure 1A, middle panel) and divide to 70-80% confluence after 72 h (Figure 1A, right panel). On the other hand, SUM149 cells grown on 3D model became connected together after 24 h (Figure 1B, left panel) and formed aggregates after 48 h (Figure 1B, middle panel). Clumps of cells simulate *in-vivo *emboli like structures were observed after 72 h (Figure 1B, right panel). The morphology acquired by SUM149 cells when seeded *in-vitro *in 3D culture model resembles *in vivo *tumor emboli of IBC patients (Figure 1C) where IBC carcinoma cells adhered together forming clusters surrounded by endothelial cells and basement membrane [10,11]. The adherence of IBC cells within lymphatic vessels forming tumor emboli was assumed to be attributed to over-expression of E-cadherin [8].

### Cytokines profile of human monocytes U937 conditioned media (U937-CM)

**Figure 2:**
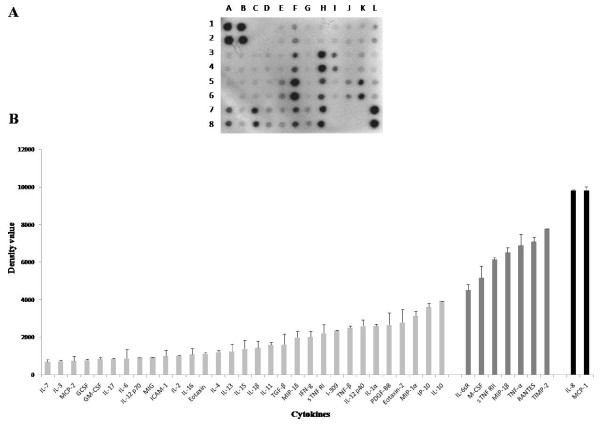
**Cytokine profile of U937-CM**. **A) **Human ChemiArray™ Human Inflammation Antibody Array III (Chemicon) assessed 40 cytokines, chemokines and growth factors, spots represent detected cytokines, chemokines and growth factors at different density levels. Detected low secreted cytokines were: Eotaxin (E_1,2_), Eotaxin-2 (F_1,2_), GCSF(G_1,2_), GM-CSF (H_1,2_), ICAM-1(I_1,2_), IFN-γ(J_1,2_), I-309 (K_1,2_), IL-1α (L_1,2_), IL-1β (A_3,4_), IL-2 (B_3,4_), IL-3(C_3,4_), IL-4 (D_3,4_), IL-6 (E_3,4_), IL-7 (G_3,4_), IL-8 (H_3,4_), IL-10 (I_3,4_), IL-11(J_3,4_), IL-12 p 40 (K_3,4_), IL-12 p 70 (L_3,4_), IL-13 (A_5,6_), IL-15 (B_5,6_), IL-16 (C_5,6_), IL-17 (D_5,6_), IP-10 (E_5,6_), MCP-2 (G_5,6_), MIG (I_5,6_), MIP-1a (J_5,6_), MIP-1d (L_5,6_), TGF-β (B_7,8_), TNf-β (D_7,8_), s TNF RI (E7,8) and PDGF-BB (G7,8). Detected moderately secreted cytokines were: IL-6 S R (f3, 4), M-CSF (H5, 6), MIP-1b (K5, 6), RANTES (A7,8), TNF-α (C7,8), s TNF RII (F7,8) and TIMP-2 (H_7,8_). While, detected highly secreted cytokines were IL-8 (H_3, 4_) and MCP-1 (F_5, 6_). **B) **Bars represent dot intensities of the measured cytokines as quantified by ImageJ software. Density values 0-4000 were considered as low secreted cytokines; 4000-8000 were considered as moderately secreted cytokines, above 8000 were considered as highly secreted cytokines. Results were representatives of 3 independent experiments.

Using cytokine antibody array analysis cytokines, chemokines and growth factors secreted by U937 were identified in U937-CM. Cytokine antibody array detected difference in density values of the assessed cytokines, chemokines and growth factors (Figure 2A). Densitometry analysis performed with ImageJ software showed that cytokines, chemokines and growth factors of low density value (0-4000) were: IL-7, IL-3, MCP-2, GCSF, GM-CSF, IL-17, IL-6, IL-12p70, MIG, ICAM-1, IL-2, IL-16, Eotaxin, IL-4, IL-13, IL-15, IL-1β, IL-11, TGF-β, MIP-1δ, IFN-γ, sTNFRI, I-309, TNf-β, IL-12p40, IL-1α, PDGF-BB, Eotaxin-2, MIP-1α, IP-10, and IL-10, respectively. Cytokines, chemokines and growth factors of moderate density values (4001-8000) were: IL-6sR, M-CSF, sTNFRII, MIP-1β, TNF-α, RANTES and TIMP-2, respectively, while cytokines and chemokine of high density value (> 8000) were IL-8 and MCP-1 (CCL2) (Figure 2B).

### U937-CM induced the expression of adhesion molecule fibronectin by SUM149 cells in 3D tissue culture models

**Figure 3:**
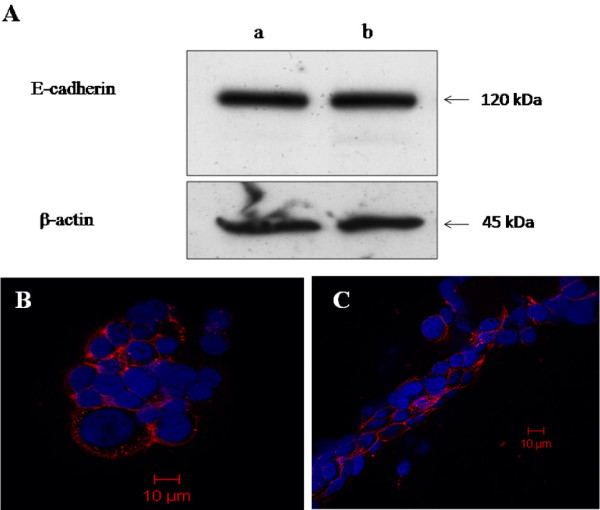
**Expression of E-cadherin by SUM149 cells**. **A) **Immunoblot analysis did not detect any difference in E-cadherin expression by control SUM149 cells gown in complete culture media (lane a) versus those grown U937-CM (lane b), β-actin was used as loading control. **B & C) **Immunostaining showed no difference in expression of E-cadherin (red) by SUM149 cells grown in complete culture media (B) versus SUM149 cells grown in U937-CM (C). While, SUM149 cells grown in U937-CM (C) migrate and form branched like structures. Nuclei were stained with DAPI (blue) and scale bar = 10 μm. Results were representatives of 3 independent experiments.

Results revealed that U937-CM induced the migration and formation of branched like structures by SUM149 cells; which was consistent with previous results of the author and colleagues [41]. The level of expression of E-cadherin by SUM149 cells grown in U937-CM, for 48 h at 37°C in humidified CO_2 _incubator, in comparison to control ones grown in complete Ham's F-12 media under same conditions was measured by immunoblot. The expression of E-cadherin was not altered by control SUM149 cells grown in complete Ham's F-12 media compared to SUM149 cells grown in U937-CM (Figure 3A). Immunocytochemistry confirmed immunoblot results which showed no difference in the level of expression of E-cadherin by control SUM149 cells grown in complete Ham's F-12 media (Figure 3B) when compared to SUM149 cells grown in U937-CM (Figure 3C).

**Figure 4:**
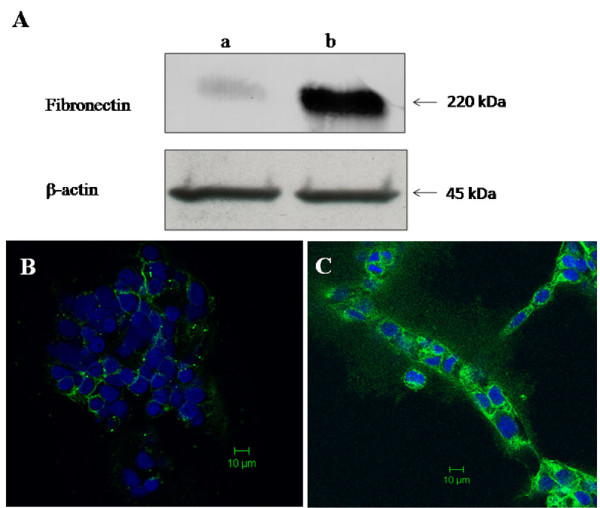
**Expression of fibronectin by SUM149 cells**. **A) **Immunoblot analysis revealed that fibronectin was weakly expressed by SUM149 cells grown in complete media (lane a) and highly expressed by SUM149 cells grown in U937-CM (lane b), β-actin was used as loading control. **B) **Immunostaining showed low expression of fibronectin (green) by SUM149 grown in complete culture media. **C) **SUM149 cells grown in U937-CM migrate and form branched like structures highly express cellular and membranous fibronectin. Nuclei were stained with DAPI (blue) and scale = 10 μm. Results were representatives of 3 independent experiments.

On the other hand immunoblot analysis showed that the adhesion molecule fibronectin, which is responsible for cell-cell and cell-matrix interaction was weakly expressed by control SUM149 cells grown in complete Ham's F-12 media and over-expressed by SUM149 cells grown in U937-CM for 48 h in 3D culture model (Figure 4A). Similarly, immunocytochemical staining of fibronectin revealed a weak expression of cellular and membranous fibronectin by control SUM149 cells grown in complete Ham's F-12 media (Figure 4B) compared to SUM149 cells grown in U937-CM for 48 h in 3D culture model at 37°C in CO_2 _incubator (Figure 4C).

### IL-8 signaling pathway is involved in fibronectin expression by SUM149 cells

**Figure 5:**
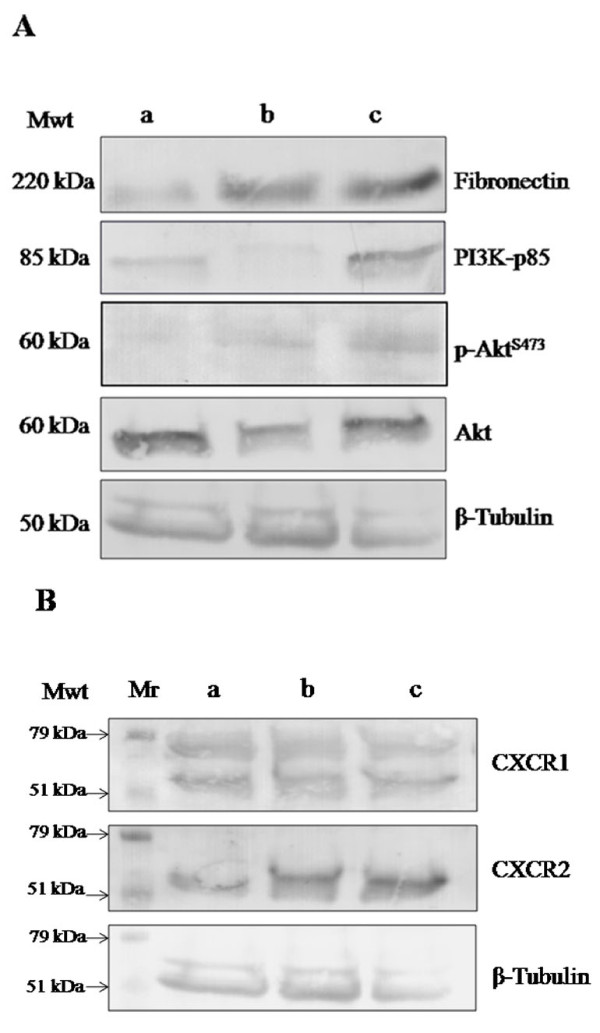
**IL-8 signaling pathway is involved in fibronectin production by SUM149 cells**. **A) **Immunoblot analysis revealed that fibronectin was weakly expressed by control SUM149 cells grown in complete media (lane a). On the other hand SUM149 cells grown in media conditioned by MCP-1 and IL-8 highly express fibronectin (lanes b and c, respectively). Immunoblot analysis of PI3K-p85 revealed that control SUM149 (lane a) and SUM149 treated with MCP-1 (lane b) weakly express PI3K-p85 while SUM149 cells treated with IL-8 showed high expression of PI3K-p85. Phospho- and total Akt were analyzed by immunoblotting. Results revealed that control SUM149 (lane a) and SUM149 treated with MCP-1 (lane b) weakly express p-Akt, while SUM149 cells treated with IL-8 characterized by more expression of p-AKT (lane c). Total Akt found to be expressed by control SUM149 (lane a), SUM149 treated with MCP-1 (lane b) and SUM149 treated with IL-8 (lane c). **B) **Immunoblot analysis showed no alteration in expression level of IL-8 specific receptors CXCR1 and CXCR2 by control SUM149 cells grown in complete media (lane a), SUM149 cells treated with recombinant MCP-1 (lane b) and SUM149 cells treated with recombinant IL-8 (lane c). Mr. represents molecular weight marker. Results were representatives of 3 independent experiments.

Treatment of SUM149 cells with recombinant MCP-1 and IL-8 induced the expression of fibronectin by SUM149 cells as detected by immunoblot analysis (Figure 5A). Since, PI3K/Akt signaling molecules found to be involved in fibronectin expression [19,20] the level of expression of PI3K, Akt and p-Akt in SUM149 cells treated with recombinant IL-8 and MCP-1 were measured. Immunoblot results showed that IL-8 induced the expression of PI3K-p85, total Akt and p-Akt by SUM149 cells. On the contrary, MCP-1 did not alter the level of expression of the PI3K/Akt signaling molecules by SUM149 cells (Figure 5A).

Since the physiological effects of IL-8 are mediated by its specific receptors CXCR1 and CXCR2, the expression of CXCR1 and CXCR2 by SUM149 cells were assessed by immunoblot. Results revealed that SUM149 cells express CXCR1 and CXCR2 regardless the treatment of MCP-1 and IL-8 (Figure 5B). The present results are consistent with other studies which showed that IL-8 specific receptors CXCR1 and CXCR2 may regulate fibronectin expression through PI3k/Akt pathway [40,43].

## Discussion

Adhesion molecules E-cadherin and fibronectin are hallmarks of epithelial mesenchymal transition (EMT), cancer invasion and motility [44]. E-cadherin expression by breast carcinoma cells was found to be regulated epigenetically via hypermethylation of the promoter region which provides cancer cell with flexibility to switch between EMT at primary tumor site and mesenchymal epithelial reverting transition (MErT) at the site of metastasis [9]. Reversion from epithelial to mesnchymal type at the site of metastasis depends on signals from the tumor microenvironment [7]. Studies postulated that IBC cells disseminate as a cluster of cells adhered together by adhesion molecule E-cadherin resulting in vascular blocking and organ failure leading to more aggressiveness of the disease [3]. On the other hand, adhesion molecule fibronectin is a mesenchymal marker regulating cell-cell and cell-matrix interactions. Fibronectin has been identified to be responsible for formation of branched like structures of salivary glands [45]. In mammary gland development and tumorigenesis fibronectin is involved in motility and branching morphogenesis [6]. Fibronectin also participates in transmitting signals from the ECM and tumor microenvironment to carcinoma cells. For instance, interaction between α5β1-integrin and fibronectin in T4-2 breast cancer cells is essential for therapeutic response [46].

Elevated inflammatory cytokines circulating in cancer patients stimulate the expression of adhesion molecules which facilitate interactions between metastatic cells and target organs [47]. Furthermore, interactions between adhesion molecules and cues from inflammatory cells within the tumor microenvironment plays a crucial role in stimulating premalignant cells to undergo malignant transformation, migration and dissemination of carcinoma cells [31,48].

Three dimensional tissue culture models are reliable tools to study IBC biology and interactions with inflammatory cells. For instance, SUM149 carcinoma cells grown on 3D matrigel culture were able to maintain E-cadherin expression essential for formation of emboli like structure similar to IBC carcinoma recognized in IBC xenograft models and IBC patients' pathological specimens [30]. Moreover, 3D culture allows cells to be adhesive to basal lamina, which may indicate the preference of IBC carcinoma cells to the connective tissue and lymphatic fluid through which they disseminate [30]. Previous studies by the author and colleagues used 3D culture showed that when SUM149 cells were directly co-cultured with human monocytes U937 or in U937-CM, SUM149 became more invasive and formed branched like structures, a mechanism that was modulated by an increase in proteolytic activity and caveolin-1 expression of SUM149 [41]. The present study emphasized the reliability of 3D tissue culture models to study the paracrine interaction between SUM149 and U937 cells. Herein, cytokines, chemokines and growth factors secreted by U937 cells in the culture media were characterized and their role in modulating the level of expression of adhesion molecules E-cadherin and fibronectin by SUM149 cells in 3D models was identified. In the present investigation U937 cells were found to secrete different levels of inflammatory cytokines. IL-8 and MCP-1 were the major highly secreted cytokines by U937 cells. Inflammatory breast carcinoma SUM149 cells seeded in media conditioned by U937 cells displayed motile phenotype and formed branched like structures associated with over-expression of adhesion molecule fibronectin, whereas no alteration in the expression of E-cadherin was detected.

Increases in the production of IL-8 and MCP-2 was found to be associated with over-expression of fibronectin by human proximal tubular HK-2 cells stimulated by TGF-β1, a mechanism mediated by connective tissue growth factor (CTGF) downstream signaling [49]. The present results disagree with Li and colleagues results which found that media conditioned by U937 did not alter expression of adhesion molecules E-cadherin or fibronectin by HK-2 cells, while direct cell-cell interaction between human proximal tubular HK-2 and U937 cells in monolayer culture induced EMT via up-regulation of intercellular adhesion molecule-1(ICAM-1), and fibronectin and down-regulation of E-cadherin by HK-2 cells [50]. The difference between these results and the present results may be due to different cell lines and tissue culture models used. In the present study, we used 3D models that mimic tumor microenvironment and allow cell-cell, cell-ECM and cell-soluble mediators interactions.

The interaction of IL-8 with its specific receptors stimulated the motility of highly metastatic human chondrosarcoma cell line (JJ012) through PI3K/Akt signaling pathway [43]. Interestingly, PI3K/Akt/mTOR signaling pathway found to augment fibronectin expression by fibroblasts [20]. The 3D cell-based assay is recommended to study the role of fibronectin in morphogenesis and neoplasia of human breast *in-vitro *culture models [6]. Increased expression of fibronectin mediates invasion and motility of colorectal cancer *in-vitro *culture [51] and is associated with positive metastatic lymph nodes *in-vivo *[52]. In mammary gland development and carcinogenesis fibronectin expression is associated with mammary cells transformation to spindle like shape, motility and formation of branching like structures [6]. The present study showed that cytokines, chemokines and growth factors secreted by U937 induced fibronectin expression and formation of branched like structures by IBC cell line SUM149. Here, U937 secreted cytokines, chemokines and growth factors induced SUM149 motility and invasion without modulating E-cadherin expression. This supports the hypothesis that IBC cells disseminate as clumps of cells adhered together by adhesion molecule E-cadherin resulting in aggressiveness of the disease [3] and suggests a role for fibronectin and IL-8 signaling pathway in IBC invasion and dissemination.

## Conclusions

The present study provides new information on the interplay of monocytes and IBC cells in the process of tumor emboli formation and spreading of IBC cells. Moreover results indicated that IL-8 may enhance motility and spreading of IBC by increasing fibronectin expression though PI3K/Akt signaling pathway.

## Methods

### Cell line and Reagents

IBC cell line SUM149 was a gift from Dr. Stephen P. Ethier, Barbara Ann Karmanos Cancer Institute, Wayne State University, Detroit, MI, USA. U937 human monocytic cell lines [American Type Culture Collection (ATCC, Manassas, USA], anti-CXCR2 and anti-CXCR2 polyclonal antibodies (From Thermo Scientific Pierce Antibodies, IL. USA) were a gifts from Dr. Bonnie Sloane's lab, Department of Pharmacology, Wayne State University, Detroit, MI, 48201, USA. Ham's F-12 media was purchased from Mediatech (Manassas, VA, USA). Monoclonal mouse E-cadherin antibody and Dispase was purchased from BD Biosciences, Inc. (San Diego, CA, USA). Monoclonal anti-fibronectin antibody was purchased from Sigma-Aldrich (St. Louis, MO, USA) monoclonal anti-human fibronectin Antibody from R&D Systems, Inc. (Minneapolis, MN, USA). PI3 Kinase p85 (PI3K-p85) Antibody, Akt Antibody and phosphorylated Akt (p-Akt) were purchased from cell signaling technology (Danvers, MA, USA). Fluorescein-conjugated affinity-purified donkey anti-rabbit IgG and normal donkey serum were purchased from Jackson ImmunoResearch (West Grove, PA, USA). ChemiArray™ Human Inflammation Antibody Array III kit was from Chemicon (Temecula, CA, USA). Cultrex^® ^Basement Membrane Extract (BME) was from Trevigen (Gaithersberg, MD, USA). Recombinant Human CCL2/JE/MCP1 and Recombinant Human CXCL8/IL8 form R&D Systems (Minneapolis, USA) were kind gift from Dr. Robert J. Schneider's lab, Department of Microbiology, New York University, New York, NY, 10016, USA. Acrylamide and nitrocellulose membranes were from BioRad (Hercules, CA, USA). Horseradish peroxidase-labeled goat anti-mouse IgG and Membrane peroxidase substrate 3, 3', 5, 5' -Tetramethylbenzidine (TMB) were purchased from Kirkegaard and Perry Laboratories (KPL), Inc. (Gaithersburg, MD, USA). Bradford Reagent for determining protein concentration was from Sigma (Sigma-Aldrich, Germany). ProSieve^® ^Color Protein Marker was purchased from Lonza (Cologne GmbH, Germany).

### Culture of SUM149 cells in monolayer and 3D models

For monolayer culture assay, SUM149 cells at density of 250 × 10^3^/ml were seeded on plastic Petri dishes in Ham's F-12 complete media and examined after 24 h, 48 h and 72 h by Zeiss Axiovert microscope (Carl Zeiss AG, Germany). To examine cell morphological changes on 3D culture, 30 mm Petri dishes were coated with 200 μl basement membrane extract BME (Culterx^®^) incubated at 37°C in CO2 incubator for 15 min till solidification. Than SUM149 cells suspension at density of 250 × 10^3^/ml was mixed with 2% BME overlaid on coated Petri dishes as described before [41]. Cells on 3D were examined microscopically after 24 h, 48 h and 72 h and morphological changes were inspected.

### Preparation of human monocytes U937 conditioned media (U937-CM)

Media conditioned by U937 cells secreted cytokines, chemokines and growth factors (designated as U937-CM) used for cytokine profiling and as conditioned media for seeding of SUM149 were prepared as was described before [53]. Briefly, U937 cells were seeded at density of 2.5 × 10^5 ^cells/ml in Ham's F12 complete media into T-75 tissue culture flasks and grown to approximately 80% confluency. Cells were collected washed twice with phosphate-buffered saline (PBS) and incubated overnight in serum-free medium. Overnight condition media were collected and centrifuged at 700 *g *at room temperature for 5 min to pellet cells. The supernatant was collected and centrifuged at 2000 *g *at 4°C for 10 min to remove cell debris. The supernatant representing U937-CM was divided into two aliquots one subjected for cytokine profiling and the second aliquot was 5-fold concentrated using AmiconUltracell10K filters (Millipore, Billerica, MA) for conditioning SUM149 culture media.

### Cytokine profiling of human monocytes U937-CM

One ml of serum free U937-CM was profiled using a ChemiArray™ Human Inflammation Antibody Array III kit that detects 40 different cytokines, chemokines and growth factors. Method was conducted as was described before [53]. Washing buffers and antibody dilutions were provided with the kit and prepared as the kit instruction guidelines. Antibody-array membrane was placed in special tray provided with the kit followed by addition of 1 ml blocking buffer for 30 min at room temperature on a horizontal shaker followed by washing. The membrane was incubated with 1 ml U937-CM and placed overnight at 4°C. After washing, the membrane was incubated with primary antibody biotin-conjugated anti-cytokines at room temperature for 2 h while shaking. Washing and incubation with HRP-conjugated streptavidin at room temperature was carried out for 2 h. After a final wash, the membrane signals were detected by adding chemiluminescence detection reagent provided with the kit and the membrane was exposed to X-ray film for different time intervals. Signal intensities representing fold differences between detected cytokines were analyzed by imageJ software (National Institutes of Health, MD, USA) as described elsewhere [54]. Relative cytokines, chemokines and growth factors levels were calculated by subtracting the background staining and normalizing density value of each cytokine, chemokine and growth factor spot to the positive control spots on the same membrane. To compare relative U937-CM detected cytokines, chemokines and growth factors the density value detection limit was divided into three ranges as follows: 0-4000 low secreted cytokines; 4001-8000 moderately secreted cytokines and above 8000 were considered as highly secreted cytokines.

### Utilizing 3D culture model to grow SUM149 cells in U937-CM

Concentrated U937-CM prepared as described above was diluted 1:5 with Ham's F12 complete culture medium containing 5% FBS then used as culture media for SUM149 cells.

To prepare 3D culture model, 30 mm Petri dishes was coated with 200 μl BME. Coated Petri dishes were incubated at 37°C in CO2 incubator for 15 min to solidify. SUM149 cell suspension at density of 250 × 10^3^/ml was mixed with 2% BME seeded as overlay culture over solidified BME and left at CO_2 _incubator for 10-15 min till cells stick as we described before [41]. Complete Ham's F-12 media were added and cells were incubated at 37°C in CO_2 _incubator. After 72 h, when SUM149 form spheroid like structures, Ham's F-12 complete media containing 5% FBS was changed in the control Petri dishes and replaced by media conditioned by U937-CM in the tested Petri dishes. Culture media were discarded after 48 h and SUM149 cells were washed several times with PBS then serum free media were added and cells were incubated at 37°C in CO_2 _incubator overnight.

### Preparation of SUM149 cell lysates

Serums starved SUM149 cells grown as control and in U937-CM were collected from BME by incubating them in Dispase solution for 2 h at 37°C in humidified CO_2 _incubator to dissolve BME. Then the cell suspension was transferred into a tube, mixed up and down several times, followed by addition of EDTA to stop the action of Dispase. Cells were centrifuged at 700 g and the pellet was washed several times and lysed in lysis buffer. Cell lysates were sonicated on ice in a 50 W Ultrasonicator five times for five seconds.

### Sodium dodecyl sulfate polyacrylamide gel electrophoresis (SDS-PAGE) and immunoblot analysis

SDS-PAGE was conducted as described elsewhere [53]. Briefly, cell lysates were reduced and separated by SDS-PAGE (12% acrylamide). Loading was performed at a concentration of 30 μg protein per well. Gels were then transferred onto nitrocellulose membranes. Immunoblot analysis was performed with primary antibodies against each of E-cadherin (1:2500), fibronectin (1:1000) and β-actin (1:1000). Followed by washing, and the addition of secondary antibody conjugated with horseradish peroxidase (1:10,000) in Tris-buffered saline wash buffer (20 mM Tris, pH 7.5, 0.5 M NaCl) containing 0.5% Tween 20 and 5% (w/v) non-fat dry milk. After washing, bounded antibodies were detected by adding TMB chromagen/substrate solution. Once the color was developed the reaction was stopped by immersing the membrane into water for 20-30 seconds.

### Immunocytochemical stain and confocal microscopy

To localize and test expression of adhesion molecules E-cadherin and fibronectin by control SUM149 cells cultured in complete culture media in comparison to SUM149 cells cultured in U937-CM, cells were seeded in 24 well plates at density of 30 × 10^3 ^cell/well. Each well was lined with a cover slip coated with 30 μl BME. After 72 h, cells were washed and media conditioned by U937 secretions were added. Ham's F-12 complete media was added to the parallel wells as controls. After 48 h the cells were washed with PBS (37°C), and then incubated overnight in serum free media. For immunocytochemical staining, cells were fixed with -20°C methanol for 5-10 min, at room temperature. Cells were permeabilized with 2% saponin in PBS, blocking was achieved by incubation with 0.5% bovine serum albumin in PBS. E-cadherin and fibronectin staining was carried out as previously described [55]. Primary antibodies used were rabbit anti-human E-cadherin (1:50) and fibronectin (1:50). Texas-Red-conjugated affinity-purified donkey anti-mouse IgG (20 mg ml^-1^) was used as secondary antibody. Negative control for immunocytochemistry was run similarly but in the absence of primary antibodies. Coverslips were mounted upside-down with antifade reagent on super frost slides. Flouresence labeled cells were detected by Zeiss LSM 510 confocal microscope (Carl Zeiss, Thornwood, NY, USA).

### Treatment of SUM149 cells with MCP-1 and IL-8

To study the role of individual cytokines MCP-1 and IL-8 on fibronectin production and relevant downstream signaling pathway (PI3k/Akt/p-Akt) SUM149 cells were seeded in monolayer as described above. After 72 h, when SUM149 form 70-80% confluence complete culture media was replaced by serum free media containing different concentrations of each of recombinant MCP-1 and IL-8 (50, 100, 150 and 200 ηg/ml). The results demonstrated that different concentrations of IL-8 and MCP-1 had similar effect on the production of fibronectin by SUM149 cells (data not shown). Thus, a dose of 200 ηg/ml of recombinant IL-8 and MCP-1 was selected as appropriate dose for treatment. SUM149 cells were grown in BME in Ham's F12 complete medium containing 5% FBS as described before. After 72 h, when SUM149 form spheroid like structures, culture media was discarded, cells were washed twice with PBS, cultured for 4 h in serum-free media with each of recombinant IL-8 and MCP-1 (200 ηg/ml). Cells were collected and prepared for SDS-PAGE and immunoblot analysis of fibronectin, PI3k/Akt/p-Akt and specific IL-8 receptors (CXCR1 and CXCR2) as described above.

## List of abbreviations

3D: three dimensional; BME: Basement Membrane Extract; CTGF: connective tissue growth factor; DAPI: 4'-6-Diamidino-2-Phenylindole; ECM: extracellular matrix; EMT: epithelial mesenchymal transition; FBS: fetal bovine serum; GCSF: granulocyte colony-stimulating factor; GM-CSF: granulocyte macrophage colony-stimulating factor; h: hour; IBC: Inflammatory breast cancer; IFN-γ: interferon gamma; ICAM-1: intercellular adhesion molecule-1; IL: interleukin; IL-12p40: interleukin-12 p40 homodimer; IL-12p70: interleukin 12p70homodimer; IP-10: interferon gamma-induced protein 10 kDa; IL-6sR: interleukin 6 soluble receptor; MCP: monocyte chemotactic protein; M-CSF: macrophage colony-stimulating factor; MErT: mesenchymal epithelial reverting transition; MIG: monokine induced by gamma interferon; MIP: macrophage inflammatory protein; MMP: metalloproteinases; PBS: phosphate-buffered saline; PDGF-BB: platelet-derived growth factor; PI3K: Phosphoinositide 3-kinase; RANTES: regulated upon activation normal T cell expressed and secreted; SDS-PAGE: sodium dodecyl sulfate polyacrylamide gel electrophoresis; TGF-β: transforming growth factor beta; TIMP-2: tissue inhibitor metalloproteinases; TNF: tumor necrosis factor; s TNF R: soluble tumor necrosis factor receptor.

## Competing interests

The author declares that they have no competing interests.

## Authors' contributions

MMM designed the study; coordinated and conducted the experiments and drafted the manuscript.
